# Graph autoencoders and community detection algorithms to improve polymorphic identification

**DOI:** 10.1093/biomethods/bpag022

**Published:** 2026-04-20

**Authors:** Carlos Patron-Rivero, Carlos Yañez-Arenas

**Affiliations:** Laboratorio de Ecología Geográfica, Unidad de Conservación de la Biodiversidad, UMDI-Sisal, Facultad de Ciencias, Universidad Nacional Autónoma de México, Sierra Papacal, Yucatán 97302, México; Laboratorio de Ecología Geográfica, Unidad de Conservación de la Biodiversidad, UMDI-Sisal, Facultad de Ciencias, Universidad Nacional Autónoma de México, Sierra Papacal, Yucatán 97302, México

**Keywords:** graph autoencoders, machine learning, artificial intelligence, polymorphism, systematics, morphology

## Abstract

Delimitation of morphotypes in cryptic lineages remains challenging because classical linear statistics and visual inspection may fail to capture complex non-linear phenotypic variations. To address this, we introduced an unsupervised machine learning framework that couples a Graph Autoencoders (GAE) with community detection algorithms to map high-dimensional morphospaces. Unlike many traditional models that treat biological specimens as independent points in feature space, our approach represents multidimensional morphological data as an interconnected network. Using a *k*-nearest neighbor graph, we captured local topological relationships and embedded them into an optimized latent space. As an empirical case study, we applied this pipeline to 484 specimens of the morphologically conservative Neotropical pitviper genus *Porthidium*, using 21 linear and pholidosis (scale counts) traits. The GAE revealed a morphological structure that showed limited concordance with current taxonomic boundaries, identifying 12 distinct morphotypes with high structural modularity (*Q *= 0.6973) but low agreement with taxonomy (normalized mutual information, NMI = 0.2812). This framework provides an objective and scalable approach for exploring phenotypic structure in complex dataset. Because it does not require prior taxonomic assignments, it may be particularly useful for investigating cryptic lineages where morphological boundaries are ambiguous. More broadly, this approach can serve as a complementary exploratory step in integrative taxonomy and evolutionary studies.

## Introduction

The integration of multidimensional datasets—from genomic sequencing to large phenotypic databases—has transformed systematics and evolutionary studies into increasingly data-intensive disciplines. These datasets offer new opportunities to investigate complex genotype-phenotype-environment interactions, but they also present analytical challenges due to their high dimensionality and potential non-linear structure [1]. Consequently, machine learning approaches have become increasingly useful for identifying latent patterns and structure in complex biological data [2].

In taxonomy and systematics, the delimitation of polymorphism (i.e. two or more distinct morphological forms within a species or lineage) has traditionally relied on human perception and linear multivariate statistics [3]. Although humans are highly capable of visual categorization [4], such assessments remain inherently subjective and may reach a perceptual limit when discerning subtle phenotypic variation. Although advanced molecular techniques, such as next generation sequencing, have helped reveal genetic divergence among lineages [5], they often overlook phenotypic integration. Phenotypic traits provide critical biological information complementary to genetic data and represent a direct outcome of evolutionary processes and environmental interactions, making them central to studies of polymorphism and species delimitation.

Particularly, cryptic lineages—where genetic divergence occurs with negligible morphological change—pose a major challenge due to the high-dimensional nature of phenotypic data and the subtle variations that distinguish closely related forms [6]. Moreover, from an evolutionary perspective, classical tools lack the sensitivity required to uncover underlying structural patterns in cryptic speciation or phenotypic convergence, thereby leaving a substantial morphological diversity understudied or entirely overlooked.

Therefore, the necessity for advanced machine learning integration becomes evident in the study of morphologically conservative and cryptic lineages. These powerful methods offer a promising pathway to overcome the aforementioned limitations, by automating the extraction of high-dimensional morphometric data from complex phenotypic datasets and revealing biologically meaningful patterns, including subtle morphotype distinctions, that remain imperceptible to human observation alone [7–9].

Specifically, deep learning architectures, such as Graph Autoencoders (GAE), represent a promising approach for evaluating patterns of phenotypic variation. Unlike approaches that represent specimens as independent observations in feature space, GAEs are designed to jointly model individual phenotypic attributes and the relational topology among samples [10]. By mapping high-dimensional morphological data into a compressed, lower-dimensional latent space, GAEs can reveal structural similarities and facilitate the identification of modular structure [11]. This graph-based representation enables a dynamic understanding of morphological disparity, explicitly addressing the complex correlations and non-linear relationships that characterize biological development and evolution. This means that, compared to commonly used dimensionality-reduction approaches (e.g. PCA, UMAP, or t-SNE), GAEs encoding this relational structure into a latent space capturing subtle variation that emerges from the connectivity patterns among specimens, variation that becomes difficult to detect with other approaches. This makes GAEs particularly suited for cryptic structure in morphospace, where meaningful biological differences may not be visible through conventional projection methods.

**Figure 1:**
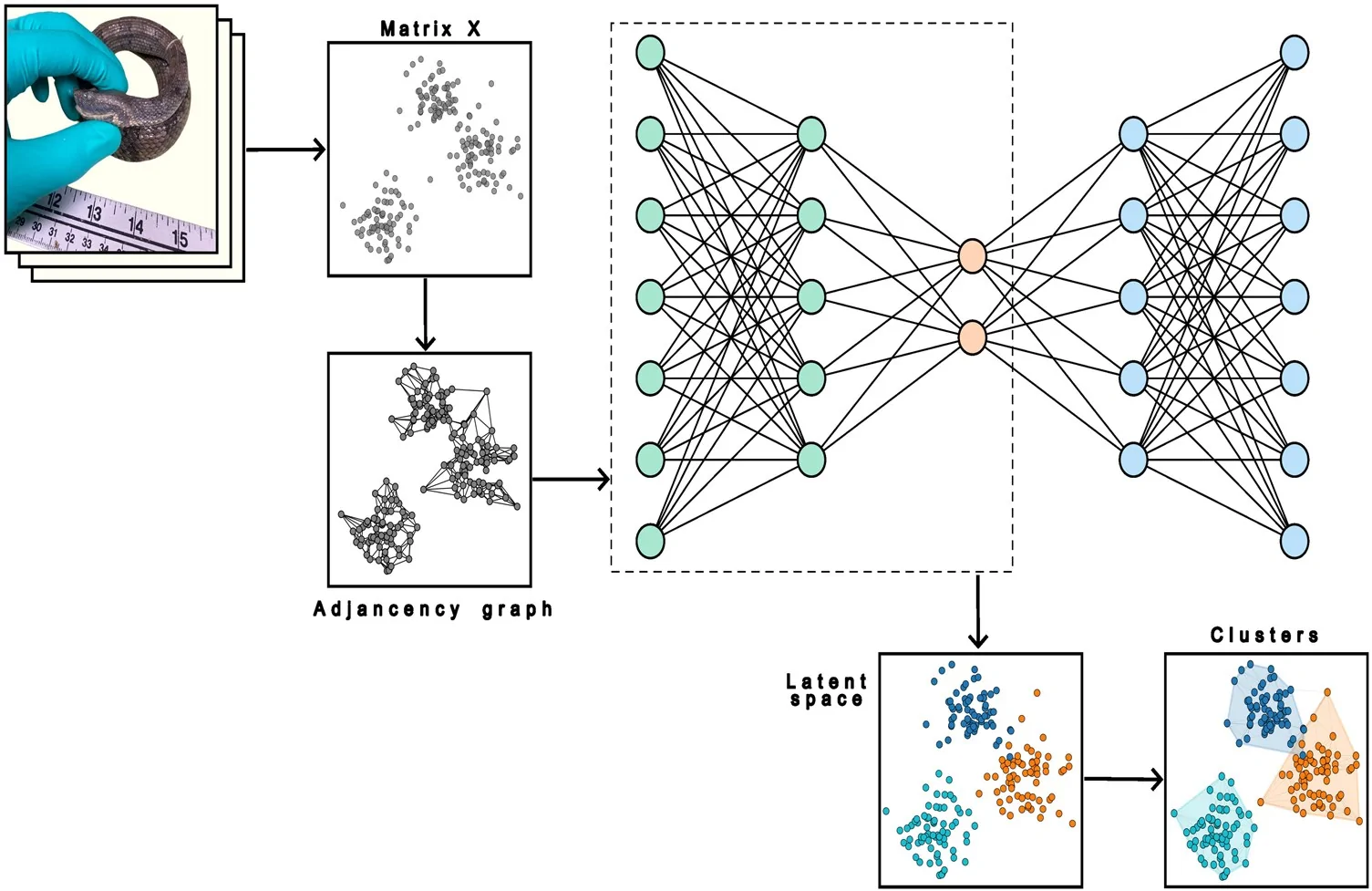
Flowchart of the GAE and the Louvain heuristic method. Given an input matrix X, a graph construction method is used to build an adjacency graph (transition matrix). After latent dimension selection, the results are projected to an embedding space to obtain cluster assignments.

Addressing the current challenges of data modeling and the critical need for reproducible, interpretable artificial intelligence in biological sciences, this study introduces a machine learning framework for the resolution of complex morphological patterns. By using the unsupervised topological learning of GAE, we provide a robust methodology to explore representations of high-dimensional morphospace of cryptic lineages. To evaluate the capacity of these computational models to detect hidden morphotypes, we employ as our empirical case study the Neotropical pitviper genus *Porthidium*, a cryptic and morphologically conservative lineage. Utilizing a multidimensional dataset of 21 morphological characters, that encompasses both linear morphometry and pholidosis (scale counts), we test the algorithm’s sensitivity to detect subtle phenotypic variations (Fig. 1). Ultimately, this approach provides a convenient method to assist in detecting patterns of morphological variation.

## Materials and methods

### Biological data

Our dataset comprises 484 *Porthidium* specimens sourced from five herpetological collections: the Field Museum of Natural History, the Biodiversity Institute and Natural History Museum of the University of Kansas, the University of Texas at Arlington Amphibian and Reptile Diversity Research Center, the Louisiana State University Museum of Natural Science, and the Colección Nacional de Anfibios y Reptiles de la Universidad Nacional Autónoma de México. The dataset includes juvenile and adult specimens with no distinction of sex.

We measured 21 morphological traits (13 morphometric and 8 pholidosis) selected for their established functional relevance to key ecological and evolutionary processes [12, 13]. These are total length (TL), snout-vent length, tail length, mid-body width, mid-body height, cloaca width, cloaca height, head length, head width, head height, distance from the head to eye, distance from the head to loreal pit, distance from the head to nostril, number of ventral scales, subcaudal scales, mid-dorsal scale rows and intersupraocular, subocular, postocular, supralabial, and infralabial scales. Comprehensive details regarding the database are openly accessible [14].

To mitigate the confounding effects of size/age, prior subsequent analysis, we applied an allometric correction. We transformed continuous morphometric traits using the natural logarithm of the ratio of the trait to total length log(trait/TL), and the meristic variables (pholidosis), using log(*x* + 1) to homogenize variances without introducing artificial size biases. Then we standardized the resulting dataset to a unit of variance to ensure equal weighting of all traits [15].

### Computational environment

We conducted all analyses using Python 3.9.13 within a Jupyter Notebook through Anaconda [16].

### Morphological graph construction

To model morphological relationships, we represented the dataset as a network where each node corresponds to an individual specimen. In a preliminary analysis, we evaluated the consistency of the modularity and the number of clusters across a range of *k* values from 3 to 10 (Supplementary Data 1). We constructed the edges using a *k*-nearest neighbors (*k*-NN) graph topology set to *k *= 4. We used this value because it optimizes modularity while maintaining the lowest standard deviation for both the modularity score and the number of inferred clusters. We symmetrized the resulting sparse connectivity matrix by taking the element-wise maximum between the matrix and its transpose, which ensures an undirected graph structure represented by the adjacency matrix A [17, 18].

To evaluate whether the number of morphotypes recovered by the GAE framework is robust to variable selection rather than an artifact of the specific set of 21 characters employed, we conducted a stratified combinatorial stability analysis. Given that the complete morphological dataset comprises 21 characters, we implemented a stratified random sampling approach, drawing up to 1000 random subsets per subset size (i.e. per number of variables included).

For each sampled variable subset, we recorded the number of communities detected by the Louvain method and the optimal number of clusters selected by the silhouette score in *k*-Means for each iteration, alongside the modularity *Q* of the resulting partition. We considered that a framework was stable if the cluster number converged on the full-dataset solution, the SD remained low across increasing subset sizes, and the proportion of subsets surpassing the modularity threshold was consistently high (Supplementary Data 2).

### Graph autoencoder architecture

We implemented an enhanced GAE to extract high-fidelity, low-dimensional representations [10]. The encoder utilizes a three-layer Graph Convolutional Network (GCN) architecture (128, 64, and *d* units) [19]. We selected this layer depth to capture high-order neighborhood interactions while explicitly avoiding the over-smoothing issues, where node representations tend to converge and lose discriminative power.

To ensure stability and efficient gradient flow, we implemented Exponential Linear Unit (ELU) activation functions and Layer Normalization [20, 21]. Furthermore, we applied a 15% dropout rate between layers. This selection was made to prevent overfitting, forcing the model to learn generalized morphological patterns rather than stochastic noise.

The decoder reconstructs the graph topology via an inner product, defined as


(1) $$\hat{A}=\sigma (ZZ^{T})$$

where $\hat{A}$ is the reconstructed adjacency matrix representing the probability of edge existence between nodes, *Z* is the latent space matrix containing the *d*-dimensional nodal representations (the compressed morphological features), and *Z^T^* is the transpose of the latent matrix. Equation (1) reconstruct the probability of edges between nodes based on the similarity of their latent embeddings.

### Optimal latent dimensionality

To address the class imbalance typical of sparse biological networks, where non-edges outnumber connections, we optimized the model using a weighted binary cross-entropy loss [22]. We evaluated the latent spaces ranging from 1 up to the 21 original morphological dimensions. Each model was trained for 1000 epochs using the Adam optimizer. We applied a learning rate decay (LRD) strategy, starting at 0.008 with 0.5 reduction every 250 epochs to promote stable convergence.

We determined the optimal latent dimensionality by evaluating four complementary structural metrics: global fidelity with the Area Under the ROC Curve (AUC); local neighborhood preservation through trustworthiness [*T*(*k*)], measuring the retention of *k*-NN in the latent space; morphological correlation, measuring the distance correlation (ρ_dist_) between the original 21 dimensions and the latent spaces dimensionality *d*; and convergence with the final reconstruction loss (binary cross-entropy, *L*_BCE_).

### Morphotype determination

To identify discrete morphological groups (morphotypes) within the genus *Porthidium*, we applied a community detection to a graph reconstructed from the adjacency matrix produced by the decoder. Once the model was fully trained, the encoder projected the morphological data into the latent space, and the decoder reconstructed a weighted adjacency matrix reflecting the relational structure learned during training. This process transitioned the analysis from continuous latent variables to categorical structural units. We converted the optimized adjacency matrix A as an undirected graph *G* = (*V*, *E*).

We implemented the Louvain method to partition the network into communities [23, 24]. Unlike traditional centroid-based clustering, Louvain is suited for biological networks because it identifies natural groupings based on connectivity density. This algorithm iteratively maximizes the modularity (*Q*), a metric that quantifies the density of connections within communities compared to a null model of random connections [25]. The modularity is defined as


(2) $$Q=\frac{1}{2m}\sum_{i,j}\left[A_{i,j}-\frac{k_{i}k_{j}}{2m}]\delta (c_{i},c_{j}\right)$$

where *A_ij_* represents the adjacency matrix between specimens *i* and *j*; *k_i_* and *k_j_* are the degrees of the respective nodes, *m* is the total number of edges; and *δ*(*c_i_, c_j_*) is the Kronecker delta function, which equals 1 if both nodes belong to the same community and 0 otherwise. Equation (2) optimization ensures that the resulting morphotypes represent robust, statistically significant partitions of the morphological network.

### Cluster validation and taxonomic concordance

We evaluated the robustness of the GAE-detected morphotypes through two primary metrics: (i) structural robustness (*Q*) where we calculated modularity values to ensure they surpassed the standard threshold of 0.3, indicating a non-random, community-rich structure in the morphological data [26, 27], and (ii) taxonomic fidelity to assess the agreement between the GAE-detected and the current taxonomic classification, for which we calculated the Normalized Mutual Information (NMI) score. This metric ranges from 0 (independence) to 1 (perfect correspondence) and quantifies the extent to which morphological disparity aligns with established species boundaries [28, 29].

Finally, to characterize the morphological identity of each detected community and identify the variables driving cluster differentiation, we applied a SHAP (SHapley Additive exPlanations) analysis [30]. We trained a random forest classifier (100 estimators) using the 12 GAE-derived cluster assignments as the response variable and the 21 standardized morphological characters as predictors. We computed SHAP values using a TreeExplainer, which decomposes the predictive contribution of each variable to the classification of every individual specimen into its additive components.

For each cluster, we quantified variable importance as the mean absolute SHAP value across all specimens, providing a per-cluster ranking of morphological predictors. Interpretation of SHAP values focused on the relative hierarchy among traits within each cluster rather than on their absolute magnitude, as the latter is an expected function of the number of classes in the multiclass classification framework. Individual contributions are necessarily partitioned across all predictors and communities [31].

### Principal component analysis baseline

To establish a comparative baseline representing classical linear multivariate statistics, we implemented a Principal Component Analysis (PCA) followed by a *k*-means clustering.

Then, we performed a PCA retaining the principal components necessary to explain 95% of the global variance. To identify discrete partitions within this linear morphospace, we applied the *k*-means algorithm, iteratively evaluating a range of clusters (*k *= 2 to 15) using 10 random initializations per run. We determined the optimal number of clusters by maximizing the silhouette score.

Finally, to allow comparison with the GAE framework, we evaluated the structural robustness of the PCA-derived clusters by calculating its modularity (*Q*) on a *k*-NN graph (*k *= 4) derived from the PCA coordinate space. We quantified taxonomic fidelity using the NMI score. We also evaluated the robustness of variable selection previously described (Supplementary Data 2).

## Results

### Morphological graph and optimal dimensionality

The construction of the *k*-NN morphological graph resulted in an undirected network comprising 484 nodes and 1184 edges. Following the symmetrization of the adjacency matrix, the network exhibited an average degree of 4.90. The overall graph density was 0.0102, reflecting sparse connectivity with potential modular structure consistent with distinct morphological groupings rather than a homogeneous continuum.

To determine the optimal latent dimensionality, we evaluated the trade-off between structural preservation and model complexity across four metrics. We identified the optimal latent dimensionality at *d *= 8, representing a stabilization point (Supplementary Data 3). At this stage, the model achieved a *T*(*k*) = 0.9014, satisfying the high-fidelity criteria for manifold preservation. *L*_BCE_ = 0.8792, representing a stability plateau where adding further dimensions resulted in only marginal error reductions. Furthermore, AUC-ROC = 0.9751, which demonstrates an accurate global reconstruction of the morphological graph. Finally, the distance correlation (*r *= −0.0203), which shows that structural variations were separated within the latent space.

### Morphotype cluster determination

**Figure 2:**
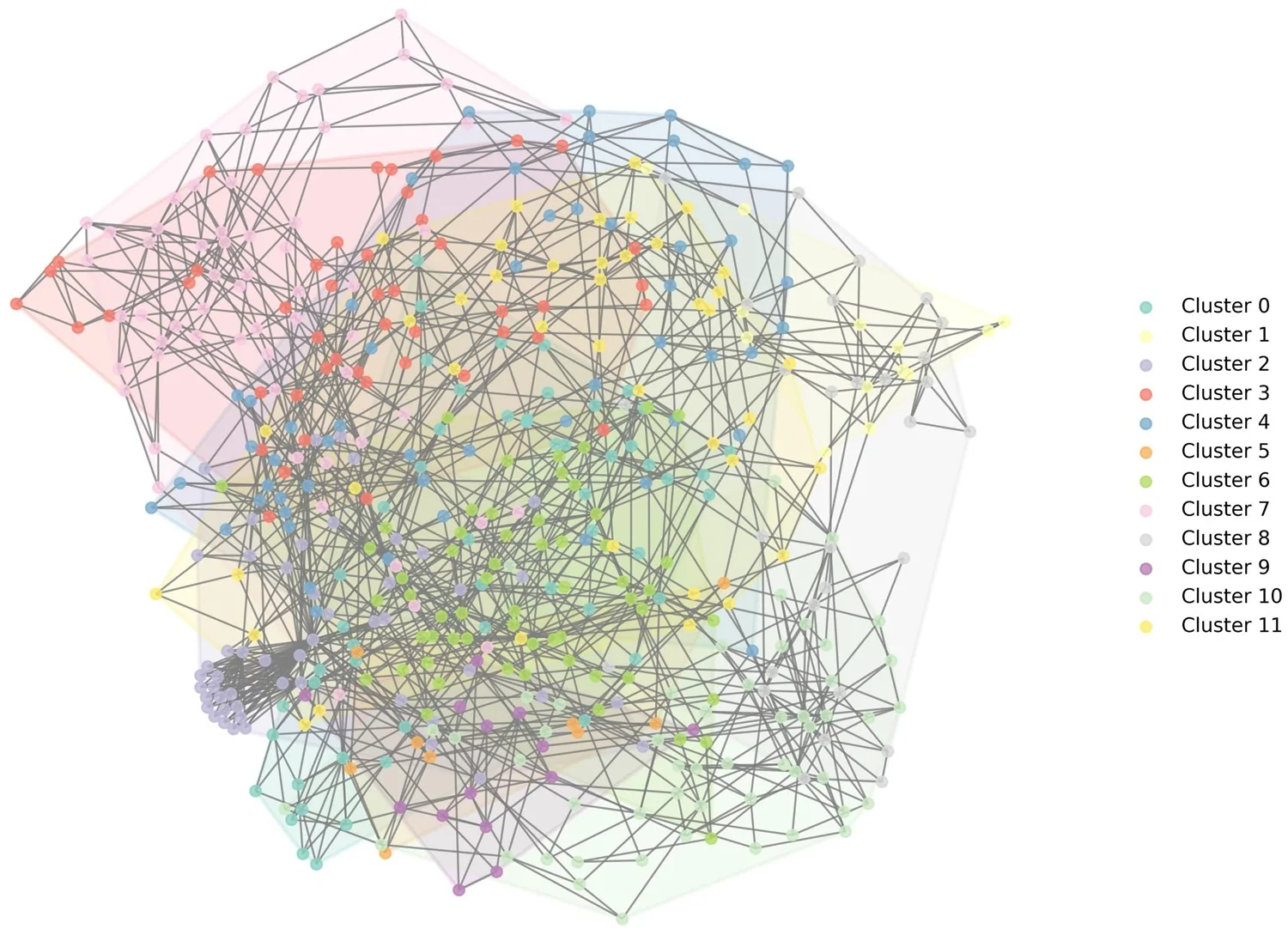
Network analysis of morphological clustering through Louvain heuristic method based on the GAE latent space (*d *= 8).

The Louvain algorithm partitioned the morphological network into 12 clusters, corresponding to the main morphotypes in the genus (Fig. 2). The analysis yielded a *Q *= 0.6973, which indicates a non-random, community-rich structure in the morphological data; with a low correspondence between morphology and current taxonomy according to NMI = 0.2812.

**Figure 3:**
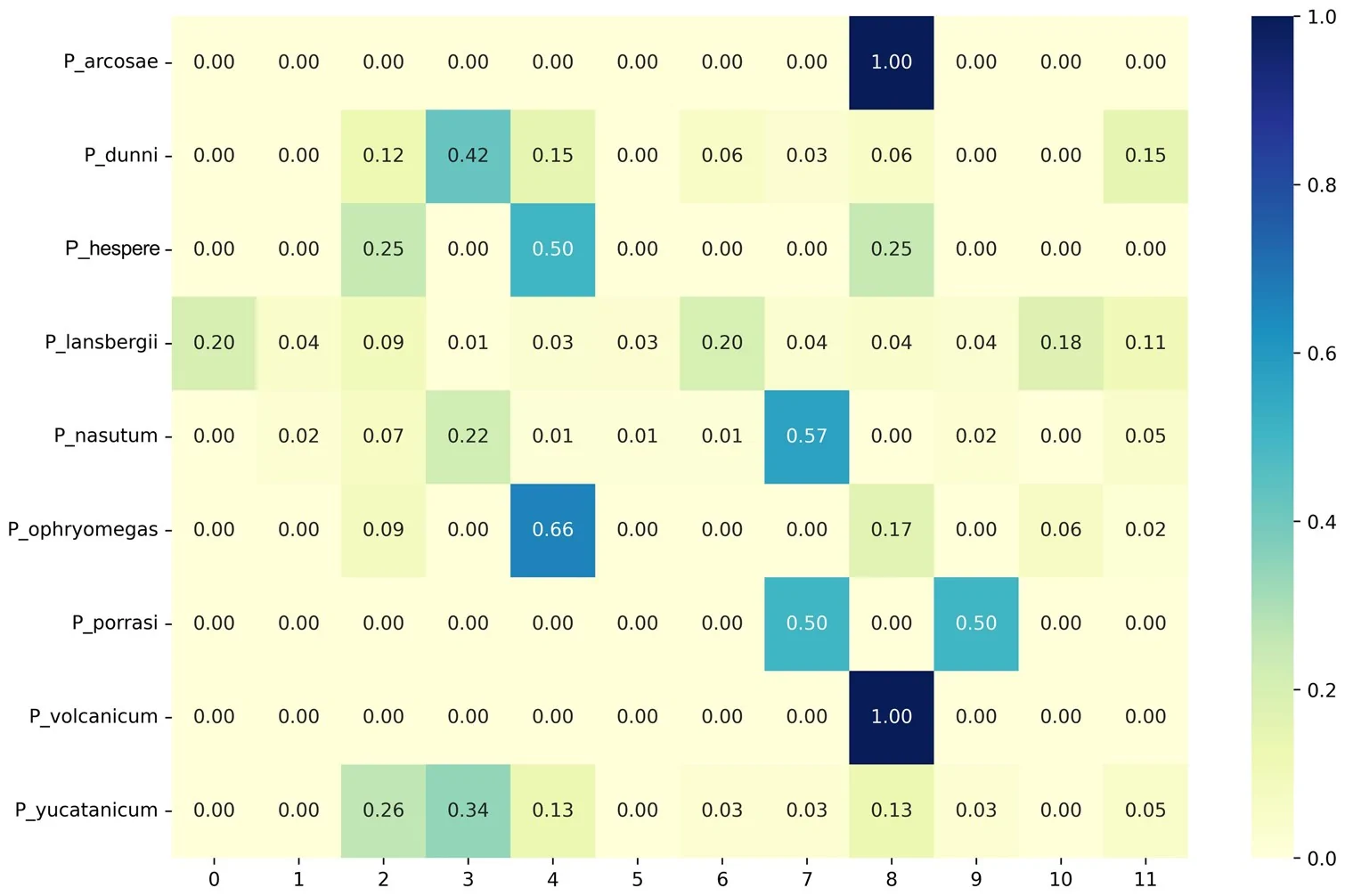
Proportion of specimens by species in each morphological cluster. Lighter yellow colors denoted lower proportions, whereas darker blue colors denoted higher proportions.

Particularly, we identified clusters ranging from containing only one species in cluster 0 (*P. lansbergii*) to seven in cluster 8. Regarding species, the results ranged from all specimens in only one cluster (*P. arcosae* and *P. volcanicum*) to *P. lansbergii* with specimens in all clusters. For *P. dunni* and *P. yucatanicum*, most of their specimens were in cluster 3, whereas for *P. hespere* and *P. ophryomegas* most specimens were in cluster 4. For *P. nasutum*, most specimens were in cluster 7. Finally, for *P. lansbergii* and *P. porrasi* most specimens were in two clusters, 0 and 6, and 7 and 9 respectively (Fig. 3).

**Figure 4:**
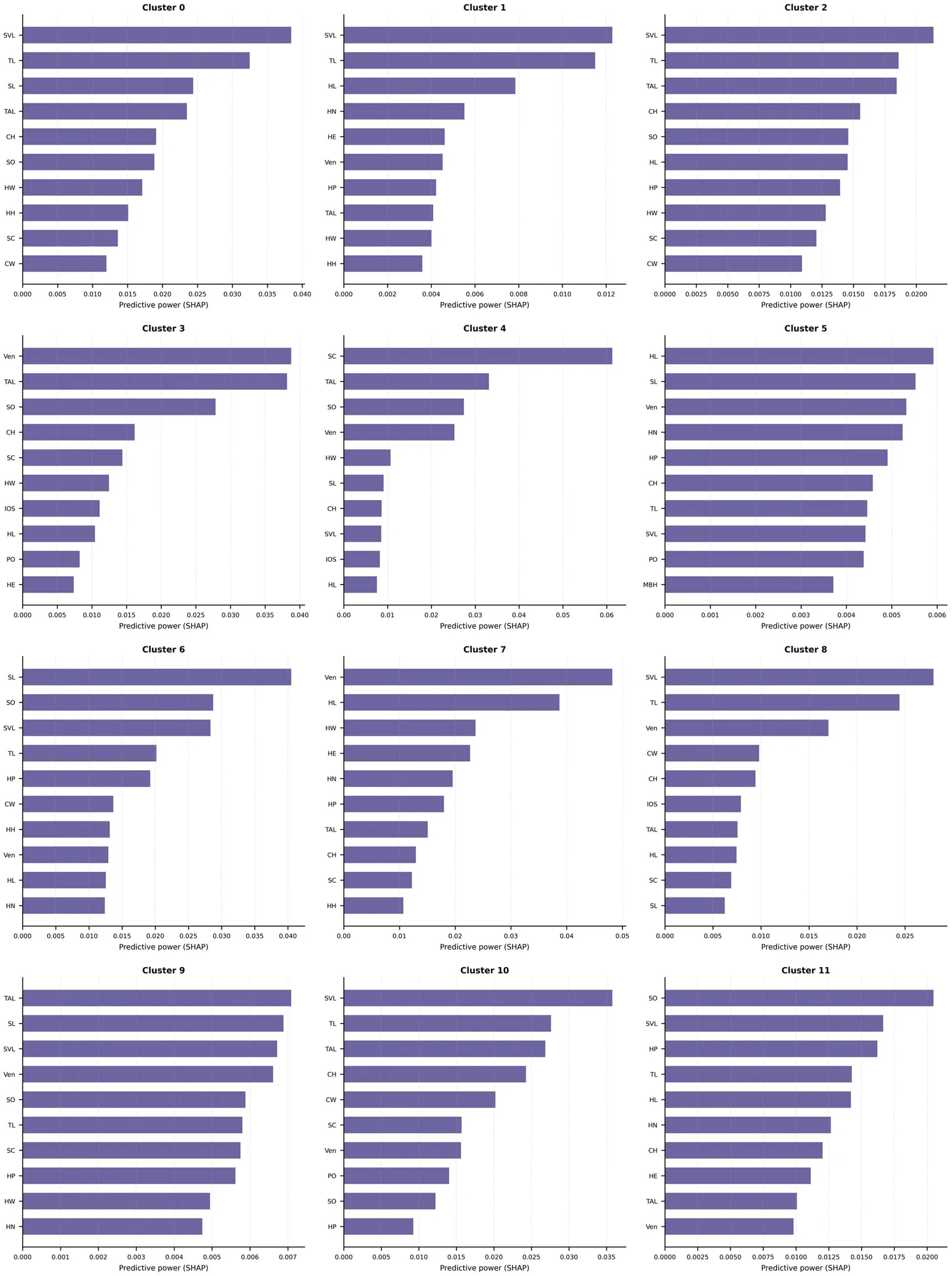
Morphological diagnosis of the 12 GAE-detected clusters based on SHAP (SHapley additive exPlanations) values. For each cluster, horizontal bars represent the mean absolute SHAP value of each morphological character across all specimens, quantifying the relative predictive contribution of each trait to cluster membership. Only the 10 most informative characters per cluster are shown, ranked in descending order of predictive power. Bar length reflects the relative importance of each trait within the cluster rather than the absolute discriminatory power, which is inherently partitioned across 12 classes in the multiclass classification framework. Trait abbreviations follow the definitions of total length (TL), snout-vent length (SVL), tail length (TAL), head length (HL), head width (HW), head height (HH), distance from head to eye (HE), distance from head to loreal pit (HP), distance from head to nostril (HN), mid-body width (MBW), mid-body height (MBH), cloaca width (CW), cloaca height (CH), ventral scales (ven), subcaudal scales (SC), mid-dorsal scale rows (MID), postocular scales (PO), subocular scales (so), supralabial scales (SL), infralabial scales (IL), and intersupraocular scales (IOS).

According to the SHAP, we identified the top 10 contributing morphological traits for each cluster, providing an objective morphological diagnosis that explicitly links each community detected in the GAE latent space to the specific phenotypic dimensions that define and distinguish it from the remaining clusters (Fig. 4).

### PCA

**Figure 5:**
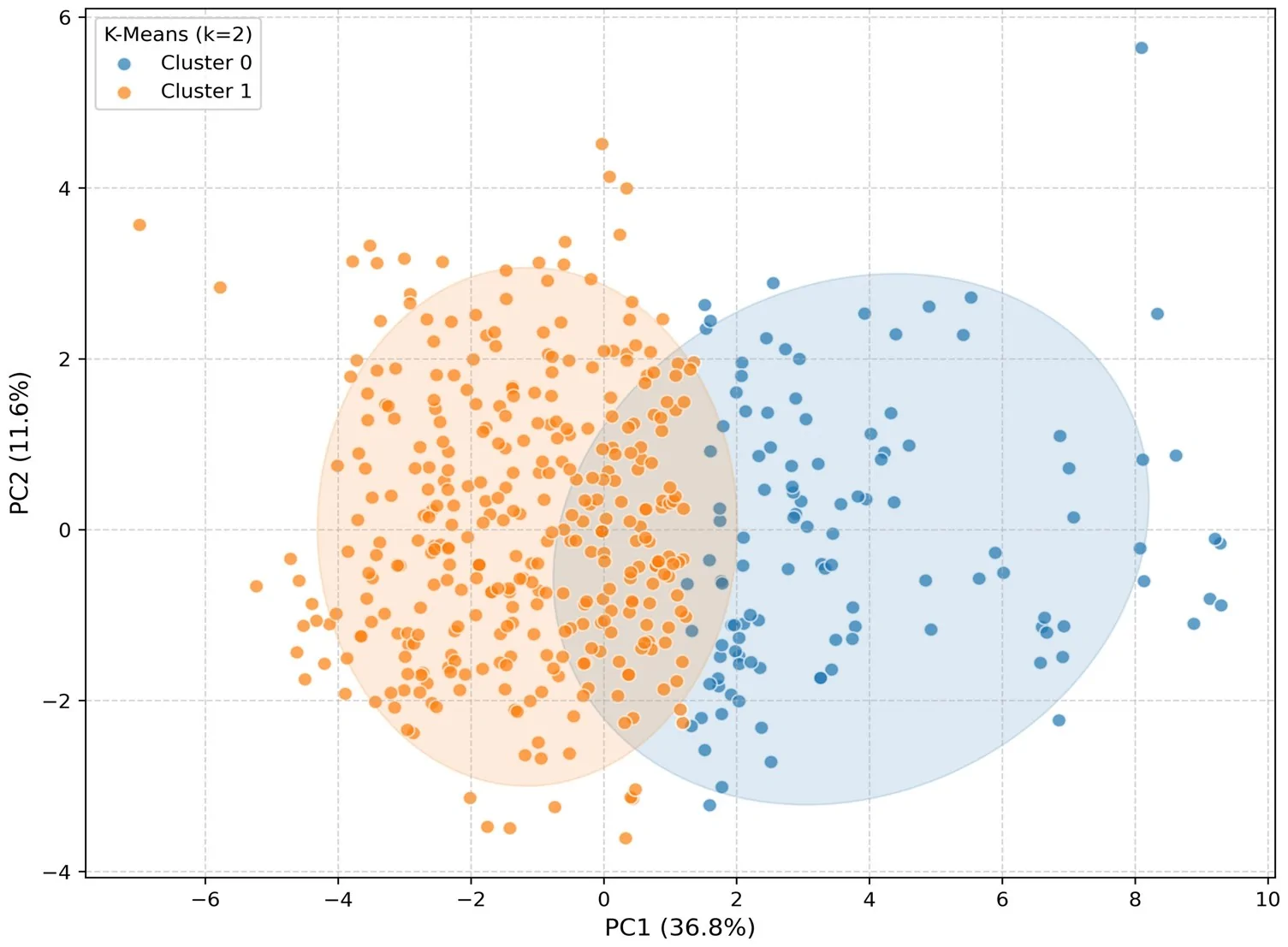
Cluster identification through principal component analysis (PCA) and *k*-means clustering.

PCA retained 14 components that together explained 95% of the variance. *k*-means clustering on these components identified two clusters with modularity *Q *= 0.2466 and NMI = 0.0355 (Fig. 5).

## Discussion

### GAE implementation

We have described the implementation of a GAE framework to map high-dimensional morphological disparity and detect cryptic phenotypic communities. We applied this methodology in the Neotropical pitviper genus *Porthidium* demonstrating that unsupervised deep learning architectures highlight a morphological structure that only partially aligns with traditional taxonomic boundaries. The robust modularity of the GAE latent space (*Q *= 0.6973), combined with the low taxonomic concordance (NMI = 0.2812), suggests that classical linear methods and human perception may overlook morphological convergence and phenotypic stasis in this lineage.

This GAE framework offers significant advantages over conventional linear analyses like PCA (Fig. 5) [32]. By processing specimens as an interconnected network, GAE simultaneously minimizes the reconstruction error and preserves the relational topology, bypassing traditional assumptions of variable independence (Fig. 2). Crucially, GAE demonstrated stability, consistently recovering 12 morphological communities regardless of the graph topology (varying *k*), the number of variables, or the specific traits selected. In contrast, although PCA also stabilized with a few variables, it identified only two clusters. This discrepancy exposes the limited capacity of linear dimensionality reduction [33, 34], demonstrating that PCA may obscure fine-scale, non-linear phenotypic structures that GAE successfully captures [35, 36].

### Biological implications

SHAP analysis reveals that morphological differentiation among GAE communities is driven by shifting combinations of linear morphometrics and pholidosis traits, rather than a single category. This suggests distinct selective pressures or developmental constraints operating differentially across the *Porthidium* morphospace (Fig. 4) [37].

Size-related variables (snout-vent length and total length) are primary predictors for several clusters, aligning with body size as a key axis for ecological divergence in squamates [38–40]. Conversely, other clusters are defined by head measurements or pholidosis, indicating that these represent independent axes of diversification. Biologically, pitviper head morphology links to venom delivery and prey handling [41, 42], while ventral and caudal scales correlate with locomotion and substrate use [43].

### Limitations and methodological considerations

Despite its robustness, the GAE framework presents limitations that warrant consideration. Deep learning architectures rely on sample size and data completeness [44–47]. Rare species or specimens could be marginalized within the latent space, potentially affecting the network modularity. Also, relying on manually curated morphological characters conceptually limits the accessible morphospace, introducing potential biases inherited from classical taxonomy [48].

In our study, the current implementation depends on 21 human-selected traits. Consequently, the algorithm can only detect structure within the variable space defined *a priori* by the investigator. Furthermore, we acknowledge that morphological sources of variation (e.g. ontogeny or sexual dimorphism) intrinsic from the dataset could influence the morphospace structure. These morphotypes must therefore be interpreted as communities within a partially observed morphospace rather than exhaustive phenotypic units. Although our stability analysis (Supplementary Data 1 and 2) confirms the framework’s robustness to trait selection within the current dataset, it cannot substitute for a broader phenotypic representation.

Finally, while the GAE objectively detects phenotypic communities, it cannot differentiate between genetic divergence or environmentally induced phenotypic plasticity [49, 50]. Therefore, these machine-learning-derived morphological networks should be integrated with genetic and ecological datasets to fully resolve the evolutionary history of the group.

### Future integration

In the broader context of systematics and evolutionary studies, this framework provides an objective and reproducible tool for deep phenotyping. The ability to identify discrete structural communities without prior taxonomic bias is essential for exploring cryptic lineages [6, 49, 51–55]. Beyond the context of *Porthidium*, the pipeline is transferable to a range of analytical domains: it can support intraspecific delimitation and resolution of polymorphic complexes; discrete morphotypes can be mapped onto phylogenies to test macroevolutionary models and estimate trait-evolution rates; metadata overlay onto the topological network enables the separation of genetic divergence from phenotypic plasticity driven by sexual dimorphism, ontogeny, or local ecology; and the integration of georeferenced occurrence data facilitates explicit tests linking morphotype distributions to environmental and spatial gradients.

However, we emphasize that this framework is designed to complement, rather than replace, traditional systematics. We recommend its use with requisite expertise and biological insight into the focal taxa or lineage, alongside active collaboration with taxonomists in future studies to ensure robust, integrative delimitation [56–58].

## Supplementary Material

bpag022_Supplementary_Data

## Data Availability

Morphological data are published and available [14]. All python codes and html are accessible and available in Github (https://github.com/PatronRiveroC/deep_learning_morphotypes).
