# Supplementary material for: Graph autoencoders and community detection algorithms to improve polymorphic identification
Source: Biol Methods Protoc. 2026 Apr 20;11(1):bpag022. doi: 10.1093/biomethods/bpag022 (PMC13171179; doi:10.1093/biomethods/bpag022)
Supplement: bpag022_Supplementary_Data [file bpag022_supplementary_data.zip › Supplementary_Data_2.pdf]

Supplementary data 2. Cluster stability results

Supplementary Figure 2. Cluster stability across variable subsets for the GAE and PCA frameworks, evaluated over independent model runs spanning all variable subset sizes (k = 2–21). a) frequency distribution of the number of clusters detected by GAE across all subsets; dashed line indicates the median. b) frequency distribution of the number of clusters detected by PCA; dashed line indicates the median. c) mean ± SD of GAE-detected clusters as a function of subset size; dotted line indicates the full-dataset reference. d) mean ± SD of PCA clusters as a function of subset size; dotted line indicates the full-dataset reference. e) distribution of modularity values (Q) across subset sizes; red dashed line indicates the $Q > 0.3$ significance threshold for non-random community structure. f) heatmap of the relative frequency (% of combinations) of each GAE cluster number detected per subset size, illustrating convergence toward $k = 12$ with increasing variable dimensionality.

Supplementary Table 2. Cluster stability summary across stratified random variable subsets for the Graph Autoencoder (GAE) + Louvain and PCA + K-Means frameworks. For each subset size (k = 2–21 variables), the mean ± standard deviation (SD), median, and modal number of clusters detected are reported across up to 1,000 independent random combinations sampled per subset size.

Cluster stability

The stratified combinatorial stability analysis, conducted across independent model evaluations spanning all possible variable subset sizes (k = 2–21), demonstrated that GAE framework consistently recovers 12 morphological communities regardless of the specific variable combination employed (Supplementary data 2). Across all subset sizes, the median and mode of the GAE-detected cluster number converged to 12 from k = 9 variables onward (12.16 ± 1.10 at k = 9, stabilizing to 12.00 ± 0.00 at k = 20), falling consistently within the central tendency of the distribution (Supplementary Fig. 2A, C). Furthermore, modularity values remained well above the $Q > 0.3$ significance threshold across all subset sizes (Supplementary Fig. 2E). In contrast, the PCA exhibited considerably higher instability at small subset sizes (8.84 ± 5.51 at k = 2), converging to its full-dataset solution of two clusters only from k = 4 (Supplementary Figure 2B, D). These results confirm that the 12 morphotypes identified by the GAE framework are not an artifact of the specific set of 21 characters employed, but rather reflect a stable structural property of the morphological network of *Porthidium*.

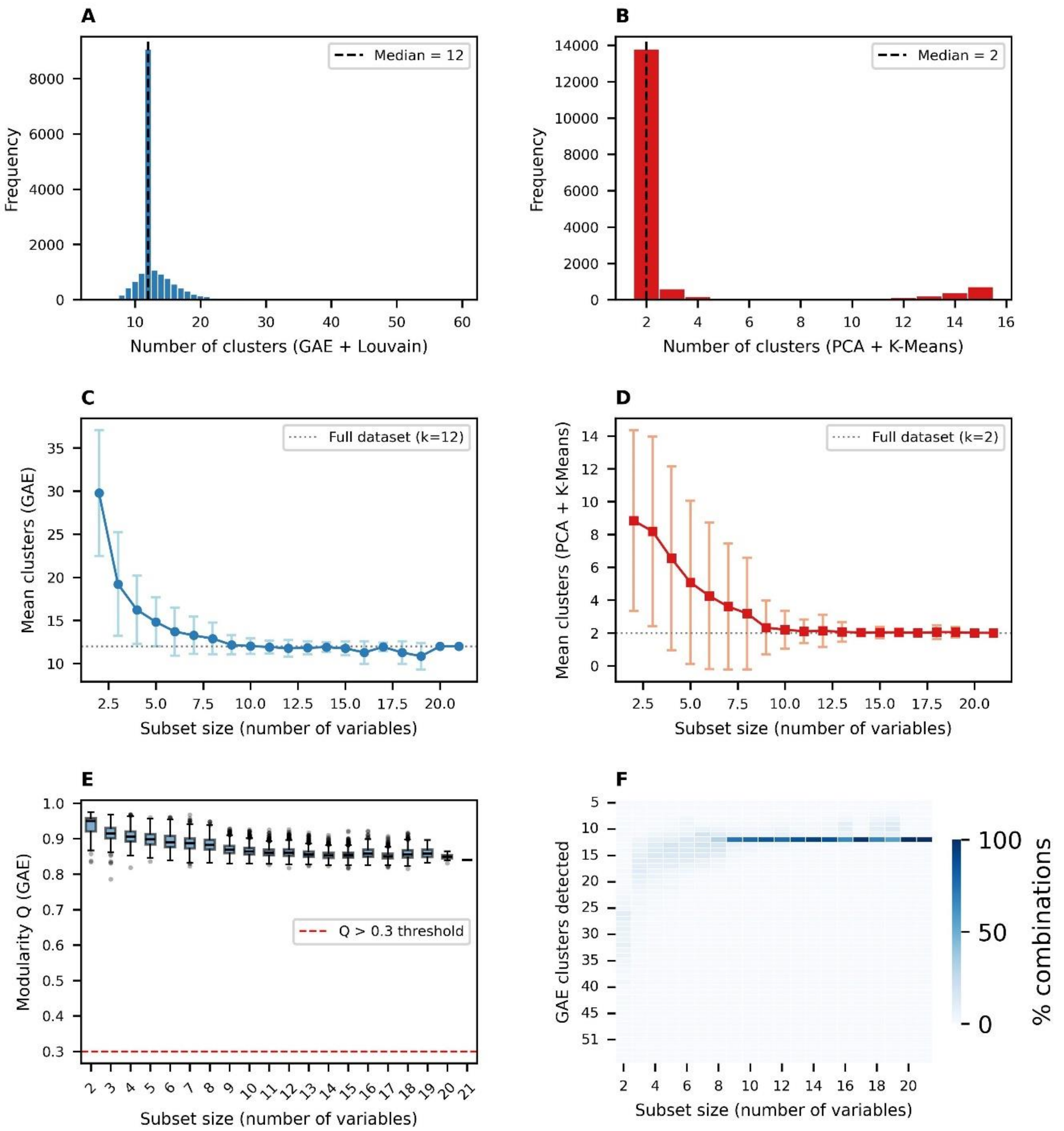

Supplementary Figure 2. Cluster stability across variable subsets for the GAE and PCA frameworks, evaluated over independent model runs spanning all variable subset sizes (k = 2–21). a) frequency distribution of the number of clusters detected by GAE across all subsets; dashed line indicates the median. b) frequency distribution of the number of clusters detected by PCA; dashed line indicates the median. c) mean ± SD of GAE-detected clusters as a function of subset size; dotted line indicates the full-dataset reference. d) mean ± SD of PCA clusters as a function of subset size; dotted line indicates the full-dataset reference. e) distribution of modularity values (Q) across subset sizes; red dashed line indicates the $Q > 0.3$ significance threshold for non-random community structure. f) heatmap of the relative frequency (% of combinations) of each GAE cluster number detected per subset size, illustrating convergence toward k = 12 with increasing variable dimensionality.

Supplementary Table 4. Cluster stability summary across stratified random variable subsets for the Graph Autoencoder (GAE) + Louvain and PCA + K-Means frameworks. For each subset size (k = 2–21 variables), the mean ± standard deviation (SD), median, and modal number of clusters detected are reported across up to 1,000 independent random combinations sampled per subset size.

	GAE				PCA			
**Subset size**	**Mean**	**SD**	**Median**	**Mode**	**Mean**	**SD**	**Median**	**Mode**
2	29.78	7.29	29	26	8.84	5.51	11	2
3	19.22	6.01	18	18	8.19	5.78	8	2
4	16.24	3.98	15	15	6.54	5.60	2	2
5	14.83	2.84	14	13	5.08	4.98	2	2
6	13.71	2.78	14	12	4.25	4.46	2	2
7	13.26	2.16	13	13	3.60	3.84	2	2
8	12.90	1.85	13	12	3.18	3.40	2	2
9	12.16	1.10	12	12	2.32	1.64	2	2
10	12.02	0.90	12	12	2.19	1.16	2	2
11	11.91	0.74	12	12	2.10	0.72	2	2
12	11.77	0.96	12	12	2.13	0.98	2	2
13	11.82	0.76	12	12	2.06	0.60	2	2
14	11.89	0.56	12	12	2.02	0.19	2	2
15	11.76	0.80	12	12	2.03	0.35	2	2
16	11.28	1.30	12	12	2.03	0.23	2	2
17	11.92	0.51	12	12	2.01	0.08	2	2
18	11.27	1.33	12	12	2.05	0.41	2	2
19	10.85	1.55	12	12	2.04	0.31	2	2
20	12	0	12	12	2	0	2	2
21	12	NA	12	12	2	NA	2	2
