# Supplementary material for: Graph autoencoders and community detection algorithms to improve polymorphic identification
Source: Biol Methods Protoc. 2026 Apr 20;11(1):bpag022. doi: 10.1093/biomethods/bpag022 (PMC13171179; doi:10.1093/biomethods/bpag022)
Supplement: bpag022_Supplementary_Data [file bpag022_supplementary_data.zip › Supplementary_Data_1.pdf]

Supplementary data 1. K-nearest neighbor's estimations.

Supplementary Table 1. Summary of k-nearest neighbor's number of clusters and modularity.

Supplementary Table 1. Summary of k-nearest neighbor's number of clusters and modularity.

	Cluster		Modularity	
**k-NN**	**Mean**	**SD**	**Mean**	**SD**
3	12.29	0.635	0.622	0.003
4	11.97	0.609	0.693	0.003
5	11.85	0.608	0.621	0.004
6	11.87	0.571	0.599	0.004
7	11.35	1.394	0.659	0.043
8	11.65	1.191	0.653	0.030
9	11.72	1.063	0.638	0.029
10	11.78	0.877	0.622	0.027
