# Supplementary material for: Graph autoencoders and community detection algorithms to improve polymorphic identification
Source: Biol Methods Protoc. 2026 Apr 20;11(1):bpag022. doi: 10.1093/biomethods/bpag022 (PMC13171179; doi:10.1093/biomethods/bpag022)
Supplement: bpag022_Supplementary_Data [file bpag022_supplementary_data.zip › Supplementary_Data_3.pdf]

Supplemental Data 3. Performance metrics and visual plot of the latent dimensionality.

Supplementary Table 3. Performance metrics for the selection of optimal latent dimensionality in the Morphological Graph Autoencoder (GAE). d = number of latent dimensions; AUC = area under the receiver operating characteristic curve. T(k) = trustworthiness score (local structure preservation). ρ_{dist} = Pearson correlation between original and latent distance matrices. L_{BCE} = final value of the weighted Binary Cross-Entropy loss function.

Supplementary Figure 3. Pairwise scatter plots of the eight optimal latent dimensions (d = 8) extracted via Graph Autoencoder (GAE). Individual specimens are colored by species to illustrate morphological clustering and overlaps within the compressed feature space.

Supplementary Table 3. Performance metrics for the selection of optimal latent dimensionality in the Morphological Graph Autoencoder (GAE). *d* = number of latent dimensions; AUC = area under the receiver operating characteristic curve. T(*k*) = trustworthiness score (local structure preservation). ρ_{dist} = Pearson correlation between original and latent distance matrices. L_{BCE} = final value of the weighted Binary Cross-Entropy loss function.

d	**AUC**	**T(*k*)**	$\boldsymbol{\rho_{dist}}$	$\mathbf{L_{BCE}}$
1	0.80	0.58	0.03	1.15
2	0.90	0.72	0.10	1.02
3	0.93	0.80	0.09	0.97
4	0.95	0.85	0.04	0.94
5	0.96	0.87	0.03	0.91
6	0.97	0.88	0.00	0.90
7	0.97	0.90	0.00	0.88
8	0.97	0.90	-0.02	0.88
9	0.98	0.91	-0.04	0.87
10	0.98	0.91	-0.05	0.86
11	0.98	0.92	-0.06	0.85
12	0.98	0.92	-0.05	0.85
13	0.99	0.92	-0.05	0.83
14	0.98	0.92	-0.08	0.84
15	0.98	0.92	-0.07	0.84
16	0.99	0.93	-0.07	0.83
17	0.99	0.93	-0.09	0.82
18	0.99	0.93	-0.08	0.82
19	0.99	0.93	-0.09	0.82
20	0.99	0.92	-0.08	0.83
21	0.99	0.93	-0.09	0.82

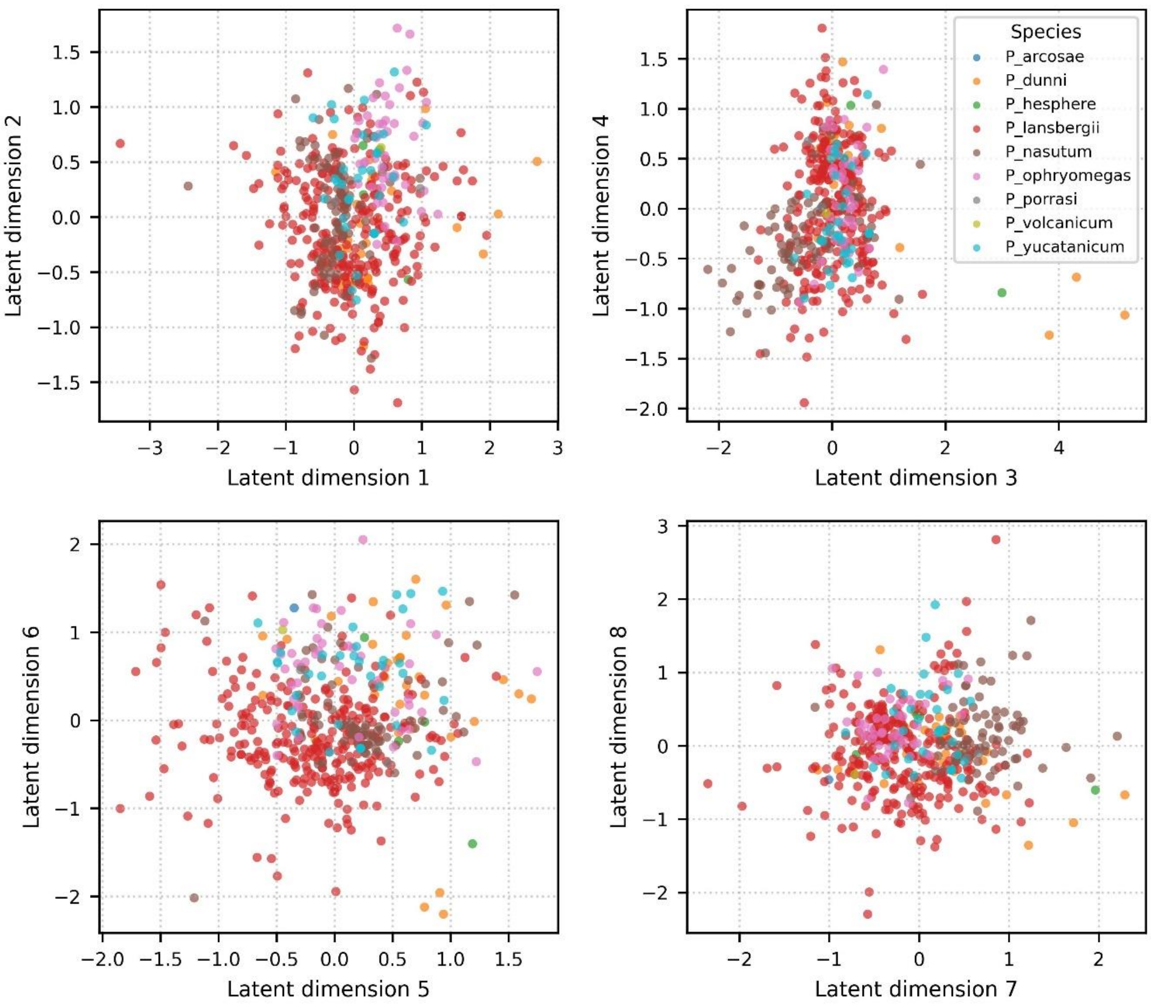

Supplementary Figure S3. Pairwise scatter plots of the eight optimal latent dimensions (*d* = 8) extracted via Graph Autoencoder (GAE). Individual specimens are colored by species to illustrate morphological clustering and overlaps within the compressed feature space.
